# Methane and carbon at equilibrium in source rocks

**DOI:** 10.1186/1467-4866-14-5

**Published:** 2013-12-12

**Authors:** Frank D Mango

**Affiliations:** 1 Petroleum Habitats, 806 Soboda, Houston, TX 77079, USA

## Abstract

Methane in source rocks may not exist exclusively as free gas. It could exist in equilibrium with carbon and higher hydrocarbons: CH_4_ + C < = > Hydrocarbon. Three lines of evidence support this possibility. 1) Shales ingest gas in amounts and selectivities consistent with gas-carbon equilibrium. There is a 50% increase in solid hydrocarbon mass when Fayetteville Shale is exposed to methane (450 psi) under moderate conditions (100°C): Rock-Eval S2 (mg g^-1^) 8.5 = > 12.5. All light hydrocarbons are ingested, but with high selectivity, consistent with competitive addition to receptor sites in a growing polymer. Mowry Shale ingests butane vigorously from argon, for example, but not from methane under the same conditions. 2) Production data for a well producing from Fayetteville Shale declines along the theoretical curve for withdrawing gas from higher hydrocarbons in equilibrium with carbon. 3) A new general gas-solid equilibrium model accounts for natural gas at thermodynamic equilibrium, and C_6_-C_7_ hydrocarbons constrained to invariant compositions. The results make a strong case for methane in equilibrium with carbon and higher hydrocarbons. If correct, the higher hydrocarbons in source rocks are gas reservoirs, raising the possibility of substantially more gas in shales than analytically apparent, and far more gas in shale deposits than currently recognized.

## Introduction

Few questions in geoscience are as interesting and controversial as the origin of methane in natural gas. And fewer go as far back in time. Evans proposed thermal cracking in 1971 to explain oil trending to methane with depth in a Canadian basin [1]. It was assumed that hydrocarbons cracked more or less randomly to smaller hydrocarbon and ultimately to methane. This premise had broad appeal, but no empirical or theoretical foundation. McNab had attempted to replicate methane generation from oil cracking in long-term cracking experiments in 1952, but failed [2]. Thermal cracking theory was nevertheless elevated to text-book status [3,4], but it was never to gain the empirical support McNab sought, and its predictive powers today are limited. It cannot, for example, explain the properties of natural gas, the thermal stability of its light hydrocarbons [5], their compositions in gas deposits [6], or their constraints to thermodynamic equilibrium [7].

Catalysis by transition metals explains these properties [5-7], and there is considerable experimental evidence supporting it. Source rocks release gas catalytically in laboratory experiments at ambient temperatures [8-10], and molecular probe experiments confirm natural catalytic activity as the source of gas in these experiments [11]. However, there is also experimental evidence against transition metal catalysis in natural gas generation [12].

Catalysis accounts for the composition of natural gas, but other questions remain troubling. Shales ingest and expel gas in the laboratory in ways contradicting classical physics. Non-classical behavior is apparent in chaotic generation curves [8], and in the generation of hydrocarbons in reverse-mass order [9]. It is also apparent in the molecular probe experiments [11]. Methane ingestion in Fayetteville and Mowry Shales reported here is another outstanding example.

The premise that methane should be independent of the solid hydrocarbons generating it could be flawed. If generation is catalytic and reversible, methane and its source would be more like carbon dioxide and calcite at equilibrium. Carbon dioxide is never independent of calcite. Ingested and expelled reversibly from CaCO_3_/CaO, its pressure increases and falls with temperature as a thermodynamic function of the equilibrium constant. Methane could similarly exist in two states, gaseous methane and solid-state methane, at equilibrium. It accounts for the results presented here and the non-classical behavior in earlier experiments.

An equilibrium hypothesis is presented in three parts. The general concept is laid out first (*Theory*). The second part is the supporting experimental evidence (*Ingestion*), and the third contains a kinetic model of the theoretical equilibrium (*Gas-Solid Equilibrium Kinetic Simulations*). A new general equilibrium model for light hydrocarbon generation summarizes the results.

## Results and discussion

*Theory* - Solid hydrocarbons storing and generating gas is hypothetical. It considers two very different reservoirs of gas in shales. The first is open porosity storing and expelling gas as a passive container in accordance with classical theory. The second does not. It is comprised of solids like pyrobitumens, and similar high-molecular weight hydrocarbons found in organic-rich shales. It is not a passive container storing hydrocarbons in solution, however. Methane could only have a very limited solubility in solid hydrocarbons, perhaps no more than a few percent by my estimates. In the current hypothesis solid hydrocarbon reservoirs are co-polymers of methane and carbon, at equilibrium. The equilibrium in reaction (1) illustrates the concept in its simplest form.

(1) $$n\;{\mathrm{CH}}_{4}+n\left(-C-\right)<=>{\left(–{\mathrm{CH}}_{2}-\right)}_{2n}.$$

The equilibrium hypothesis and supporting evidence are discussed below. Here, we consider the origin of solid hydrocarbons, also referred to as ‘carbon pools’. ‘Methane’ is used throughout to illustrate relationships for simplicity. All references to ‘methane’ or ‘CH_4_’ should apply to the higher light hydrocarbons as well, although methane is the primary focus here.

The existence of solid hydrocarbons in source rocks is not new. They have been recognized by organic geochemists for decades. Generating gas from higher hydrocarbons necessarily generates a carbon deposit to balance hydrogen. It is the text-book explanation for the origin of pyrobitumen, for example, a ubiquitous organic mineral in sources rocks with H/C ratios ranging from about .5 to 1.6 [4]. Pyrobitumens are typically rich in transition metals [13,14] and have recently been cited for catalytic activity in methane generation [15]. Solid hydrocarbons have been proposed as catalysts resembling activated carbon in the decomposition of higher hydrocarbons to gas [16]. Solid hydrocarbon pools in this model can be pyrobitumen, or any other organic solid containing a catalyst that generate gas. They are both catalysts for generating methane and reservoirs for storing it.

Solid hydrocarbons are not uncommon in transition metal catalysis. The hydrogenation of carbon monoxide to methane over nickel, ‘methanation’, is an outstanding example [17]. The intermediates to methane are distributed in pools of solid hydrocarbons associated with nickel. The nature of the carbon polymer in methanation is unclear except that it is a co-catalyst, it is unsaturated in hydrogen and it is not graphite. It is like the carbon pool proposed here, some carbon polymer between saturate hydrocarbon CH_2_ and graphite CH_0_. The activated carbon suggested by Alexander et al. [16] in the catalytic generation of natural gas is another relevant example.

How the proposed equilibrium reaction might proceed mechanistically is discussed in *Gas-Solid Equilibrium Kinetic Simulations*, below. We are only concerned here with overall conversion. Methane generation feeds carbon into a pool of hydrogen-unsaturated carbon, into pyrobitumen, for example.

Since source rocks possess natural catalytic activity and generate catalytic gas in the laboratory [11], we shall assume that natural methane is catalytically generated and that the catalyst resides in pools of solid hydrocarbons. Because most catalytic reactions are reversible and approach equilibrium over time, we also assume that methane generation is reversible and should bring methane and solid hydrocarbons into equilibrium over time.

Reaction (2) illustrates a general equilibrium between methane and solid hydrocarbons.

(2) $$x\;{\mathrm{CH}}_{4}+{\mathrm{CH}}_{y}<=>C_{x+1}H_{y+4x}$$

There is a 46% mass difference in methane equivalents between a solid hydrocarbons with compositions CH_0.5_ and CH_1.6_ , for example, a typical range for pyrobitumens [4]. Thus, CH_0.5_ can consume 46% of its mass in methane generating CH_1.6_ if methane exists in equilibrium with solid hydrocarbon.

However, there is nothing to suggest that methane actually *reacts* with solid hydrocarbons generating new compounds. It is not enough that methane merely dissolves in CH_y_. In that case, x in reaction (2) would be insignificant and the equilibrium in (2) meaningless.

The proposed equilibrium is purely hypothetical, but testable. Do solid hydrocarbons in source rocks consume extraordinary amounts of light hydrocarbons in ways distinguishable from simple adsorption and solution?

*Light Hydrocarbon Ingestion-* The experimental challenge is to distinguish gas addition to the solid hydrocarbons in source rocks from classical gas addition to ordinary solid hydrocarbons. There are several classical ways light hydrocarbons can add to heterogeneous materials like source rocks. They can be adsorbed on surfaces, go into liquid solutions, or into polymer solutions, for example. There is no chemical change in adsorption and solution, however. They are first-order reactions in which rates of addition are proportional to concentrations: Rate = k*(X), where (X) is the concentration of free hydrocarbon X and k is the first-order rate constant. Because hydrocarbons do not compete for surface sites or positions in solution, transfer rates and solubilities are typically independent of other hydrocarbons. Butane has about the same water solubility in helium as it has in methane, for example [18].

Assume that hydrocarbons add to the solid hydrocarbons in source rocks differently. Hydrocarbon X adds to some receptor [···] generating the adduct [X], a process referred to here as ‘ingestion’. X disappears by second-order kinetics at rates proportional to X and [···]: Rate = k*(X)*[···]. However, in contrast to adsorption and solution, hydrocarbons compete for [···]. Thus rates of ingestion are not independent of other hydrocarbons.

We can distinguish between ingestion and either adsorption or solution through competitive addition. If solid hydrocarbons are mere solvents, hydrocarbon X will go into solid-solution at rates proportional to concentrations of X, *independent* of some other hydrocarbon Y. If, however, X reacts with [···], and Y competes with X for [···], X will go into the solid at rates inversely proportional to Y.

Do hydrocarbons compete in their addition to solid hydrocarbons?

To put this question to experimental test, two vials were charged with identical amounts of Mowry Shale and an equal molar mixture of ethane, propane, *iso*-butane and *n*-butane (C_2_-C_4_). Both vials were heated at 75°C for 200 hours. One (Vial *A*) was diluted with argon by 50% five times over the 200 hours and the other (Vial *B*) was diluted with equal amounts of methane five times. The only difference in the two reactions was dilutions with argon in one (Vial *A*) and dilutions with methane in the other (Vial *B*). Thus, C_2_-C_4_ gas concentrations in each vial diminished equally with each argon and methane dilution. Concentrations over time should be about the same in *A* and *B* if Mowry Shale is passive and only removes C_2_-C_4_ hydrocarbons by adsorption or solution. If it removes them by ingestion, and C_1_-C_4_ hydrocarbons compete for [···], C_2_-C_4_ concentrations in Vial *A* should fall progressively below those in Vial *B*.

Another vial (C) was charged with beach sand with a thin coating of n-octadecane (~ 1%) to assess adsorption and solution. The C_2_-C_4_ hydrocarbon mixture was added and the vial was heated and diluted with methane as was Vial *B*.

If Mowry Shale consumes hydrocarbons classically through adsorption and solution exclusively, the ratio of sums ∑ (C_2_-C_4_)_A_ / ∑ (C_2_-C_4_)_B_ should remain constant over time. If, however, Mowry Shale *ingests* C_2_-C_4_, the ratio of slums ∑ (C_2_-C_4_)_A_ / ∑ (C_2_-C_4_)_B_ should progress to zero over time.

**Figure 1:**
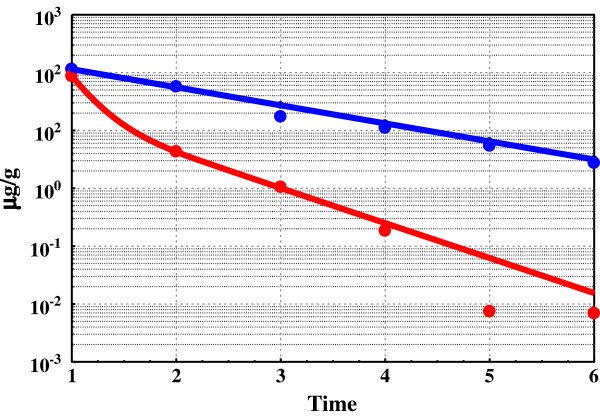
**n**-**Butane ingestion in Mowry Shale from argon and methane.** Concentrations of hydrocarbons in vials over time (in arbitrary units) are in Table 1. Blue solid dots are gas concentrations of n-butane in methane (Vial B) and red solid dots are gas concentrations in argon (Vial A). The blue line traces the concentration of n-butane diminishing by 50% with each dilution. It is not the regression line for the Vial B data. The red line is the DED regression line for the Vial A data. Data points below the blue line reflect n-butane ingestion into Mowry Shale.

**Figure 2:**
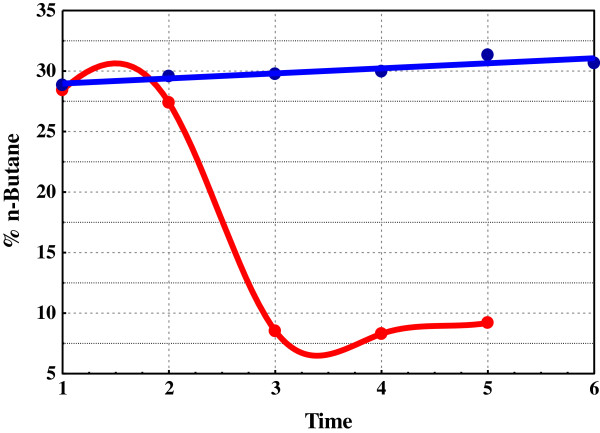
**% wt n**-**Butane in C2**-**C4 in Vials A and B.** % Concentrations of hydrocarbons in vials over time (in arbitrary units) are in Table 1B. Red: Vial A (argon); Blue: Vial B (methane).

Hydrocarbon gas concentrations (C_2_-C_4_) over time in the three vials are shown in Table 1. The differences between Vials *A* and *B* over time are dramatic. Mowry Shale consumed 163 μg C_2_-C_4_ g^-1^ in argon (Vial *A*) compared to under 30 μg g^-1^ in methane (Vial *B*). Figure 1 shows the decline in n-butane gas concentrations over time in *A* and *B*. Concentrations of n-butane in *B* fall exponentially consistent with sequential 50% dilutions, but% composition of C_2_-C_4_ remains essentially constant over time (Table 1B). The blue line passing through the *B* data is not the regression line for that data. It is the dilution line indicating where the data should plot if only dilution were lowering n-butane gas concentrations. Hydrocarbon concentrations in *A* fall sharply below the blue dilution line progressing to zero over time signaling almost total ingestion. Moreover, n-butane was *selectively* withdrawn from Vial A. Percent n-butane (C_2_-C_4_) fell sharply in *A* and remained essentially constant in *B* (Figure 2). Concentrations at termination were 16 μg g^-1^ in *B* and 0.064 μg g^-1^ in *A*, a 250-fold difference.

**Table 1:** **Hydrocarbon ingestion**, **Mowry Shale**, **75**°C, **200 hours**

	**Weight Concentrations in gas over time**					
**VIAL A**	**1**	**2**	**3**	**4**	**5**	**6**
μg C2/g	83.993	13.629	4.720	1.239	0.114	0.028
μg C3/g	101.548	11.542	3.113	0.693	0.036	0.010
μg iC4/g	124.533	13.942	4.059	1.032	0.065	0.020
μg nC4/g	86.679	4.290	1.051	0.184	0.007	0.007
**VIAL B**	**1**	**2**	**3**	**4**	**5**	**6**
μg C2/g	69.046	32.556	8.803	5.489	2.623	1.595
μg C3/g	93.766	44.174	12.430	7.606	3.249	1.558
μg iC4/g	119.893	59.328	19.538	12.789	5.971	3.032
μg nC4/g	114.537	57.104	17.266	11.075	5.403	2.734
**VIAL C**	**1**	**2**	**3**	**4**	**5**	**6**
μg C2/g	106.474	54.830	12.063	7.282	4.088	
μg C3/g	149.244	73.379	10.149	5.855	3.118	
μg iC4/g	191.697	92.384	10.637	6.208	3.464	
μg nC4/g	177.661	83.205	3.061	1.749	1.080	
	**Percent wt Compositions in C**_**2**_-**C**_**4 **_**over time**					
**VIAL A**	**1**	**2**	**3**	**4**	**5**	
% C2/g	**21.17**	**31.40**	**36.47**	**39.36**	**51.16**	
% C3/g	**25.59**	**26.59**	**24.05**	**22.00**	**16.12**	
% iC4/g	**31.39**	**32.12**	**31.36**	**32.80**	**29.38**	
% nC4/g	**21.85**	**9.88**	**8.12**	**5.83**	**3.34**	
**VIAL B**	**1**	**2**	**3**	**4**	**5**	
% C2/g	**17.38**	**16.85**	**15.17**	**14.85**	**15.21**	
% C3/g	**23.60**	**22.87**	**21.42**	**20.58**	**18.84**	
% iC4/g	**30.18**	**30.71**	**33.66**	**34.60**	**34.62**	
% nC4/g	**28.83**	**29.56**	**29.75**	**29.96**	**31.33**	
**VIAL C**	**1**	**2**	**3**	**4**	**5**	
% C2/g	**17.03**	**18.05**	**33.59**	**34.52**	**34.79**	
% C3/g	**23.88**	**24.15**	**28.26**	**27.76**	**26.54**	
% iC4/g	**30.67**	**30.41**	**29.62**	**29.43**	**29.48**	
% nC4/g	**28.42**	**27.39**	**8.52**	**8.29**	**9.19**	

Sample preparation and experimental procedures are described elsewhere [10]. Each 5 ml. vial was charged with 1 g shale and gas composed of a 2 ml. mixture of ethane, propane, isobutane, and n-butane, equal volumes, and about 2 ml. of either methane (Vials B & C) or argon (Vial A). 2 ml. gas was removed and analyzed 5 times over 200 hours and replaced with argon (Vial *A*), or methane (Vials *B & C*). Concentrations are gas concentrations in the vials prior to each dilution calculated from the concentrations in the 2 ml. samples removed with each dilution.

C_2_-C_4_ concentrations in Vial C fell over time proportional to their molecular weights consistent with solution in n-octadecane. The ratio of *iso*-butane/n-butane approached equilibrium (~ 3 at 75°C) consistent with acid-catalyzed isomerization promoted by the mild acidity of beach sand [19]. The equivalent reaction in Vial B showed no change in *iso*-butane/n-butane, or in the composition of C_2_-C_4_. Thus, Mowry Shale in *B* showed no evidence of isomerization activity or adsorption under the reaction conditions. We conclude from this that the solubility of hydrocarbons in Mowry Shale by solution in liquid and solid hydrocarbons is relatively insignificant under these conditions.

Mowry Shale in argon thus consumed C_2_-C_4_ hydrocarbons vigorously and selectively at 75°C over 200 hours. The same shale in methane totally rejected the same C_2_-C_4_ hydrocarbons under the same conditions.

*Methane ingestion-* Fayettville Shale was exposed to nine sequential cycles of methane pressurization and exhaustion at 100°C. Vessels filled with shale were pressurized (450 psi), sealed, and slowly vented. The shale released only trace amounts of C_2_ and higher hydrocarbons during exhaustion. There was no evidence of free methane in the shale after these experiments. Heating vented samples in Argon at 100°C produced only trace amounts of methane and higher hydrocarbons, substantially less than the original sample.

**Figure 3:**
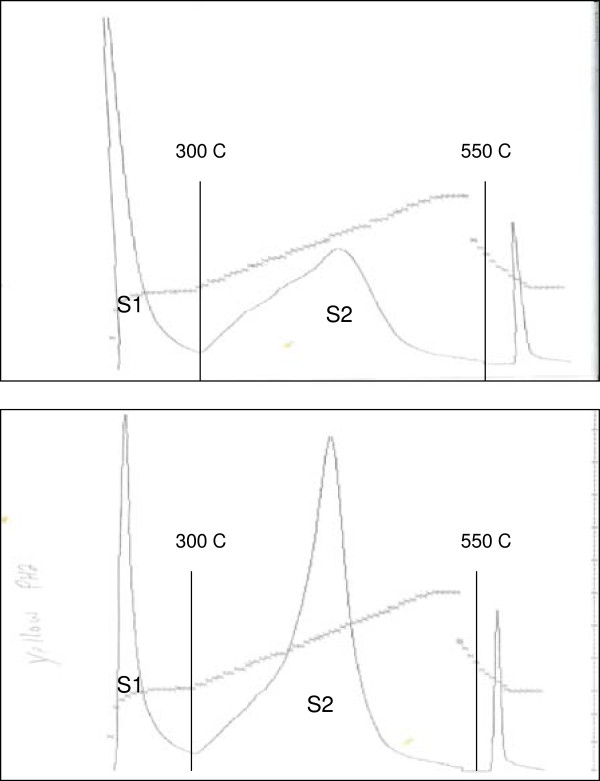
**Rock**-**Eval signals for Fayetteville Shale before and after exposure to methane.** Upper panel is the FID trace for the starting shale and the lower panel is the FID trace for the same shale exposed to 100% methane (Table 2).

Methane was indeed consumed by Fayetteville Shale, but not as free methane. It appeared in the solid hydrocarbons, in the Rock-Eval S2 peak, not in the free hydrocarbon S1 peak (Figure 3 and Table 2). Remarkably, the shale returned to its original Rock-Eval composition when exposed to the same exhaustion experiments in 2% methane. Thus, the solid S2 hydrocarbons in Fayetteville Shale had increased in mass by about 50% on exposure to high methane partial pressures and decreased in mass by 50% when exposed to low methane partial pressures. Samples with increased solid hydrocarbon mass showed no evidence of free methane either at 300°C in Rock-Eval analysis, or at 100°C for one hour in our analysis.

**Table 2:** **Rock**-**Eval analysis of Fayetteville Shale before and after exposure to methane 450 psi**, **100**°C

**Samples**	**S1**	**S2**	**S3**	**TOC**	**Tmax**
Starting	4.2	8.3	0.27	3.35	446
100% CH_4_	3.67	12.45	0.28	3.69	443
2% CH_4_	3.56	7.97	0.25	3.35	443

Shale was prepared for analysis by grinding in argon to 60 mesh. Three aliquots were treated as follows: Aliquot 1: analyzed directly by Rock-Eval. Aliquot 2: Rock-Eval analysis after 9 exposures to methane (450 psi, 100°C, 10 min.). Aliquot 3: Rock-Eval analysis after 9 exposures to methane (450 psi, 100°C, 10 min.) followed by 5 exposures to 2% methane in helium (450 psi, 100°C, 10 min.). Samples in sealed brass containers were pressurized to 450 psi at 100°C for 10 minutes in each exposure, and slowly vented (1 minute) to atmospheric pressure. Samples showed no evidence of adsorbed methane after sequential exposures to methane; standard gc analysis: 1 g shale in 5 ml. Ar, 100°C, 1 hr. S1 = mg g^-1^ volatile hydrocarbons, < 300°C; S2 = mg g^-1^ solid hydrocarbon cracking, 350 – 550°C; S3 = mg CO_2_ g^-1^; TOC =% organic carbon. Values of starting shale are averages of triplicate analyses with sd: S1 ± 0.3; S2 ± 0.3; TOC ± 1.4; TMAX ± 3. Values for products are single analyses, although one (100% CH_4_) was verified in a second analysis. Rock-Eval analyses by GeoMark Research, Humble, Texas.

Fayetteville Shale thus consumes methane at 100°C, nearly doubling its S2 mass. Mowry Shale consumes n-butane selectively in preference to ethane, propane, and *iso*-butane. In excess methane, methane is consumed to the exclusion of butane. Shales therefore consume light hydrocarbons by competitive addition to receptors generating high molecular weight hydrocarbons consistent with the proposed equilibrium between methane, carbon, and higher hydrocarbons (Reaction 1).

*Gas-Solid Equilibrium Kinetic Simulations -* Consider a source rock with free gas of concentration (C_x_), where C_x_ is a light hydrocarbon containing x carbon atoms. It is in communication with solid hydrocarbon with the capacity to generate (C_x_) from some catalytic intermediate [C_x_] where [···] denotes the concentration of open catalytic sites, the receptors noted above. [C_x_] forms through reaction (3) from [C_n_], a high molecular weight hydrocarbon bonded to an active site, for example (CH_2_)_n_-M where n > > x, an intermediate discussed elsewhere [10].

(3) $$\left[C_{n}\right]+[\cdots ]<=>\left[C_{x}\right]+\left[C_{n-x}\right]$$

We shall assume [C_n_] and [C_n-x_] in reaction 3 are indistinguishable when *n* is large (n ~ n-x), and that [C_n_] is therefore a constant in the kinetics of [C_x_] generation. Gas generation proceeds through reaction (4), a reversible reaction. [···] bonds selectively to various hydrocarbons, and only to hydrocarbons.

(4) $$\left[C_{x}\right]<=>\left(C_{x}\right)+[\cdots ]$$

A hydrogen balance is ignored throughout this scheme. All hydrocarbons in brackets are unsaturated, C_n_H_2n_, and those in parentheses are saturated, C_n_H_2n+2_. Thus, reaction (3) is hydrogen neutral. Reaction (4), however, is not, and should be ([C_x_] + H_2_ = (C_x_) + [···]) [10]. We assume for simplicity that hydrogen is in excess and that it moves easily from the pool to [C_x_] generating (C_x_) and from (C_x_) to the pool generating [C_x_].

Reactions (3) and (4) are within a closed system, a sealed source rock, for example. Gas escapes confinement through reaction (5).

(5) $$\left(C_{x}\right)=>C_{x}$$

Reaction (6) summarizes the kinetic steps interconverting free gas and solid-state gas as symbolized by [C_x_] and [C_n_].

(6) $$\left[C_{n}\right]+[\cdots ]<=>\left[C_{x}\right]<=>\left(C_{x}\right)+[\cdots ]$$

The rate of [C_x_] generation is proportional to [···]*[C_n_]. Because [C_n_] is constant and [···] becomes constant over time (steady-state), *the rate of gas generation from solid hydrocarbon is constant over time*. It will continue generating [C_x_] and free gas (C_x_) until their concentrations become sufficiently high to promote reverse reactions at rates equal to forward reactions. At that point, reaction (6) is at steady-state (equilibrium). (C_x_) and [C_x_] are then at their maximum concentrations, and [···] is at its minimum concentration. This is the state of a sealed (closed) source rock in the subsurface. We are interested in the dynamics of that system when the rock is opened, and reaction (5) is significant. How will (C_x_) decline over time when the system is at equilibrium and the rate of reaction (5) exceeds the rate of generation (reaction 3)?

Three possibilities are considered in the following kinetic simulations. The first is classical first-order expulsion without generation or equilibrium of any kind. The kinetic model thus contains only free, in-place gas (C_x_). [C_n_], [C_x_], and [···] are all zero. In the second, (C_x_) and [C_x_] interconvert at equilibrium, but [C_n_] does not generate [C_x_]; gas generation does not attend gas release, in other words. In the third, reaction (6) is fully operative, two reservoirs of gas, [C_x_] and (C_x_), at equilibrium, are depleted while [C_x_] is generated at a constant rate from [C_n_].

**Figure 4:**
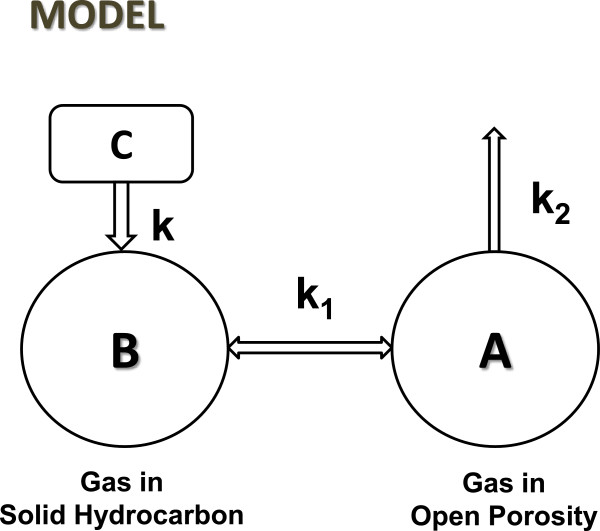
**Schematic diagram of kinetic model simulating gas generation from gas**-**solid equilibrium. *A*** and ***B*** are reservoirs charged with native gas, (C_x_) is in ***A*** and [C_x_] is in ***B*** (reaction 8). Reservoir ***C*** resides in ***B***. Open catalytic sites [···] reside in ***C*** and ***B***, unspecified in diagram. Gas passes reversibly between reservoirs as indicated by first-order kinetics (k_1_ = 0.07) and by second-order kinetics (k_-1_ = 0.007) for generation of [C_x_]. Gas passes to the surface from ***A*** by first-order kinetics controlled by rate constant k_2_ (0.2). Catalytic gas is generated in ***C*** from [C_n_] at a constant rate controlled by second-order rate constant k (0.0004), considered irreversible in this simulation. Generated gas passes directly into ***B***. Each reservoir is filled with an initial quantity of gas, assumed non-depletable in ***C*** (100), and depletable by first-order kinetics in ***A*** ((C_x_) = 200) and ***B*** ([C_x_] = 200). The initial value of [···] was assumed 5, with all concentrations unitless. Reaction 8 was simulated by iterating kinetic steps by first or second-order changes until concentrations of [···], [C_x_], and (C_x_) were constant (steady-state). For example, the concentration of [C_x_] at iteration ii was: [C_x_]_*ii*_ = [C_x_]_*i*_ + (k*[ ]_i_*[C_n_]) + (k_-1_*(C_x_)_i_*[···]_*i*_)-(k_1_*[C_x_]_*i*_). All concentrations were similarly calculated at each iteration.

**Figure 5:**
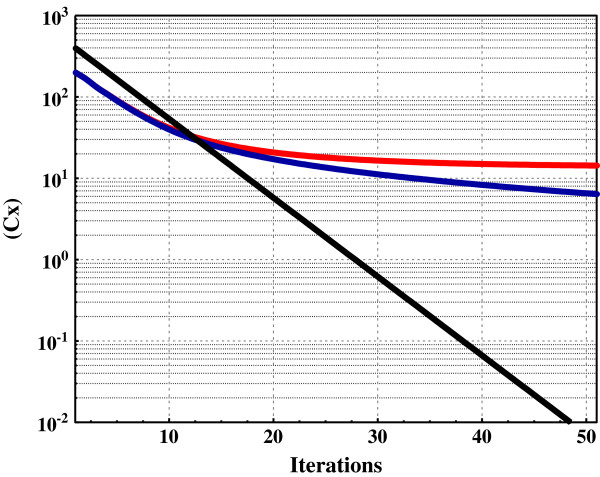
**Kinetic simulation of gas**-**solid equilibrium.** The kinetic scheme in Figure 3 was simulated in 50 iterations with the following restrictions. ***Black Curve***: Reaction 8 where [C_n_] = [C_x_] = 0. ***Blue Curve****:* Reaction 8 where [C_n_] = 0. ***Red Curve****:* Reaction 8. Starting concentrations: [···] = 5; [C_n_] = 100 (constant); [C_x_] = 200; (C_x_) = 200 (400 in first curve). Rate constants: 0.0004: [C_n_] = > [C_x_]; 0.07: [C_x_] = > (C_x_); 0.007: (C_x_) = > [C_x_]; 0.2: (C_x_) = > C_x_. The curves represent 50 first and second-order iterations. For example, the concentration of (C_x_) on iteration *ii* is: (C_x_)_ii_ = (C_x_)_i_ + (.07*[C_x_]_i_) – (.007*(C_x_)_i_) – (.2*(C_x_)_i_). Equations for lines (t = time): *black*, (C_x_) = 500 exp(-.223 t) *blue*, (C_x_) = 224 exp(-.243 t) + 27.3 exp(-.029 t) *red*, (C_x_) = 223 exp(-.243 t) + 15.5 exp(-.045 t) + 12.5.

Each possibility is treated assuming equal rate constants and equivalent intermediate concentrations. Figure 4 displays a schematic of the reactions used in the kinetic simulations. A small fraction of gas in reservoir *A* was removed by first-order kinetics in each iteration, ∆(C_x_) = (0.2*(C_x_)), and all other concentrations altered accordingly as described in Figure 3. Thus, ∆(C_x_) over iterations simulates expulsion rates over time. ∆(C_x_) is henceforth referred to as ‘rate’ denoted R and iteration as ‘time’ (t). Figure 5 shows the decline curves, R vs. t, for three hypothetical rocks. In the first (no catalytic activity) R declines exponentially (ED) as expected, thus describing the straight line on log scale. In the equilibrium models, R declines by double exponential decay (DED) without generation and by double exponential decay with a constant (DED1) with generation, (equation 7), where R_t_ is the expulsion rate at time t, (R)_i_ is the initial expulsion rate for free gas (C_x_), [R]_i_ is the initial expulsion rate for solid hydrocarbon gas [C_x_], and C is the constant for rate of generation from [C_n_].

(7) $$R_{t}={\left(R\right)}_{i}e^{-\mathrm{at}}+{\left[R\right]}_{i}e^{-\mathrm{bt}}+C$$

**Figure 6:**
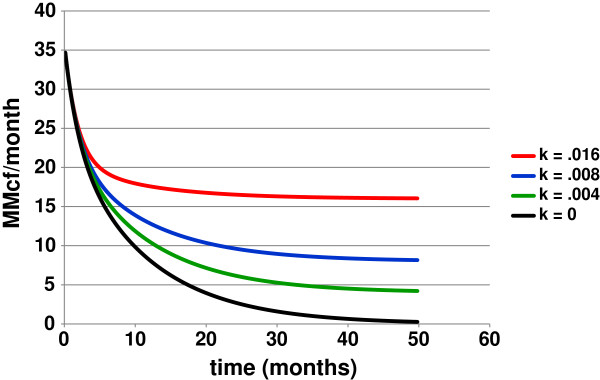
**Effects of generation on double exponential decay curves from Gas**-**Solid Equilibrium Kinetic Model.** The kinetic model in Figure 3 (modified units) generates the family of DED1 curves shown with zero-order rate constants for generation from k = 0 to k = 0.016 ([C_n_] = > [C_x_]).

Figure 6 shows DED1 curves with different values of C reflecting different levels of generation attending production. Therefore, rates of production from source rocks releasing gas in equilibrium (reaction 6) should decline by DED1 reflecting free gas declining exponentially, solid-state gas declining exponentially, and generated gas at a constant rate C.

**Figure 7:**
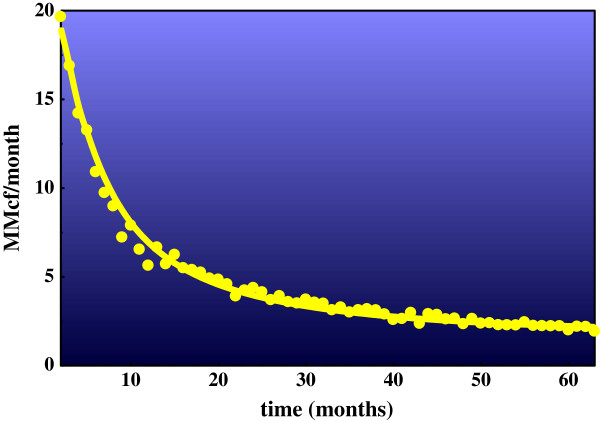
**Production decline data for Miller Heirs 1**-**10H well producing from Fayetteville Shale.** The Millers Heirs well is in the Arkoma Basin, Arkansas, Lease # 383264348; January 2006 - August 2011. The line passing through the production data is the decline curve calculated from the kinetic model in Figure 3 with the following rate constants and initial concentrations (63 iterations): k = 0.00012; k_1_ = 0.1; k_-1_ = 0.005; k_2_ = 0.16; [C_n_] = 100 (constant); [C_x_] = 250; (C_x_) = 90; [···] = 5. The rate constants and starting concentrations in Figure 3 were hand-adjusted to these values to approximate the general shape of the Miller Heirs data. The line is not a best-fit line or the DED1 regression line to the data. The respective DED1 regression equations are: Miller Heirs data (R^2^ = 0.99): R = 20.7 e^-0.266t^ + 6.71 e^-0.039t^ + 1.50. Model, Blue line: R = 16.0 e^-0.197t^ + 6.60 e^-0.0526t^ + 1.95.

*Gas production Miller Heirs well, Fayetteville Shale -* Many gas wells decline classically, by simple exponential decay, but there are notable exceptions mainly in low porosity-permeability wells including unconventional wells [20-24]. Figure 7 shows typical non-classical decay for a well producing from a source rock, in this example the Miller Heirs well producing from Fayetteville Shale. The line passing through the data is the DED1 line calculated from the kinetic model in Figure 5. It is not a best-fit equation line to the data. Showing the theoretical curve superimposed on field data in this way illustrates the remarkable fit between theory and field data. Regressing Miller Heirs data to DED1 gives an exact fit (R^2^ = 0.99), while regressing the data to single exponential decay gives a substantially poorer fit, R^2^ = 0.84. The constant C in the DED1 regression equation was significant throughout production, accounting for 37% of produced gas at 25 months.

Regressing the same data to double exponential decay without a constant (DED, eq. 7 where C = 0) gives an equally strong correlation (R^2^ = 0.99), however. It is therefore impossible to evaluate the constant C from the data fit to DED1 in Figure 7. If the data has genuine linearity – two sources of gas declining exponentially and one constant - then any DED equation that fits the data between time 0 and *n* will necessarily underestimate the data beyond *n*. The DED line will fall exponentially while the data approaches the constant C. Therefore, the test for linearity (C) lies beyond *n*, where the respective regression curves predict the future.

**Figure 8:**
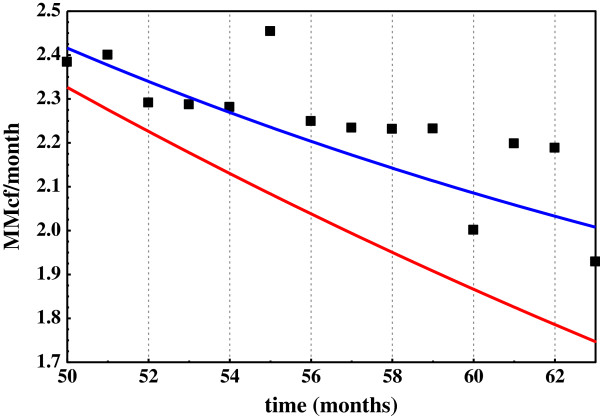
**Production data for Millers Heirs wells beyond 50 months with regression lines for DED and DED1 between 0 and 50 months.** The data in Figure 6 were regressed to double exponential decay with a constant C (DED1) and without a constant (C = 0) (DED) between 0 and 50 months. Red line: R = 21.4 e^-0.25t^ + 7.0 e^-0.022t^. Blue line: R = 20.8 e^-0.263t^ + 6.68 e^-0.0364t^ + 1.33.

The Miller Heirs data were regressed between times 0 and 50 months by DED and DED1. The two regressions gave similar curves with high degrees of correlation to the data between these time limits (R^2^ ~ 0.99). However, only the DED1 equation predicts the Miller Heirs data beyond 50 months (Figure 8). The DED line fall sharply below the Miller Heirs data. It pojects exponential decline while the data describes a largely linear rate of decline beyond 50 months. Figure 8 leaves little doubt about the dimensions of decline in this well. There are clearly three, and DED1 (eq. 9) describes them very well. DED1 is thus a property of Miller Heirs production data and the gas-solid hydrocarbon equilibrium model as Figure 7 so clearly illustrates.

## A New light hydrocarbon generation model

Few question the biological pedigree of higher hydrocarbons (biomarkers) in petroleum [3,4]. Their carbon structures are precise fits to the carbon skeletons of bio-precursors. Light hydrocarbons between C_1_ and about C_10_ are different. They do not resemble biological parents [25], and they display distinctive patterns in composition not seen in the higher hydrocarbons. Methane through butane (C_1_-C_4_) are constrained to thermodynamic equilibrium [7,10] and the hexanes and heptanes (C_6_-C_7_) display invariant compositions [26,27], for example. A proportionality between (n-C_6_*i-C_7_) and (n-C_7_*i-C_6_), perhaps the highest correlation yet reported for hydrocarbons in crude oils (R^2^ = 0.99), illustrates their extraordinary compositional order [27].

It is in this context that methane-solid hydrocarbon equilibrium must be weighed. With methane in equilibrium with ethane and propane [7], it should come as no surprise that it would be in equilibrium with the solid hydrocarbons generating it. In fact, there is a strong possibility that all light hydrocarbons form along the same path, with light hydrocarbons and solid hydrocarbons at equilibrium. It is illustrated in a new model for light hydrocarbon generation presented here. The model unifies otherwise disparate elements of light hydrocarbon chemistry: natural gas at thermodynamic equilibrium, invariance in the higher light hydrocarbons, ingestion, and gas generation during production. It does this by bringing light hydrocarbons and solid hydrocarbons into equilibrium through catalytic intermediates [C_x_] and [···]:

$$\left[\mathrm{Cn}\right]+[\cdots ]<=>\left[\mathrm{Cx}\right]<=>\mathrm{Cx}+[\cdots ]$$

[C_x_] represents the intermediate shaping product structures and compositions. Metathesis of [C_x_] brings C_1_ to C_4_ to equilibrium [10] and molecular rearrangements of [C_x_] bring C_5_ to C_7_ isomers to invariance [26,27]. Ingestion, which links methane to [C_x_] and thus to solid hydrocarbons, is the critical step in the proposed methane-solid hydrocarbon equilibrium. Mowry and Fayetteville Shales ingest light hydrocarbons in substantial amounts and with high selectivity. Mowry Shale in argon with open receptors ingests C_2_-C_4_ hydrocarbons vigorously, and the same shale in methane with pacified receptors totally rejects the same hydrocarbons under the same conditions (Figures 1 and 2). Generation and storage is expressed in the model through: [C_n_] + [···] < = > [C_x_]. It accounts for the linearity in double exponential decay decline curves (Figures 7 & 8). Thus, each component of the general equilibrium model has empirical support.

Most catalytic reactions are reversible, and approach thermodynamic equilibrium over time (residence time). It therefore follows that C_1_-C_4_ hydrocarbons residing in closed source rocks over geologic time will be at molecular and isotopic thermodynamic equilibrium, and steady state with respect to compositional change. However, once the rock is opened and old hydrocarbons escape, new hydrocarbons will be generated and their residence times can be on the order of hours. C_1_-C_4_ could then be removed from equilibrium and the C_6_ and C_7_ hydrocarbons, typically constrained to constant compositions including metastable equilibria [26-28], displaced from these compositions as well. In other words, molecular and isotopic compositions of hydrocarbons generated at steady state can be distinct from hydrocarbons generated at pre-steady state. Our research has focused on hydrocarbons generated at steady state over geologic time. There is less know about pre-steady state because there have been fewer opportunities to find and analyze pre-steady state products. However, we have encountered oils from conventional reservoirs with bizarre C_6_ and C_7_ distributions consistent with pre-steady state kinetics at the onset of oil generation [29]. Unconventional production from source rocks offers opportunities for finding similar pre-steady state hydrocarbons. Compositions could transition between states in early production, capturing molecular and isotopic biases only rarely seen in conventional reservoirs. The laboratory for finding evidence of that transition is in the field, from wells producing oil and gas from source rocks, where the transition might be captured and analyzed.

## Conclusions

Production rates declining by DED1 is not in itself significant. But, the coincidence of theory and data in Figure 6 is. It implicates gas-solid hydrocarbon equilibria in the production of gas from Fayetteville Shale.

Ingestion gives the hypothesis additional and independent support. First, it is unprecedented. Light hydrocarbons react with solid hydrocarbons in source rocks under conditions where they do not react with ordinary solid hydrocarbons. Methane, perhaps the least reactive hydrocarbon known, disappears in Fayetteville Shale at 100°C leaving no trace of CH_4_. It does not emerge in RockEval analysis in the S1 peak at 300°C, but later as some higher hydrocarbon in the S2 peak at 443°C (Table 2). Methane thus becomes part of the solid hydrocarbon ingesting it: C_1_ + [···] = > [C_1_]. The reactions between C_1_-C_4_ hydrocarbons and Mowry Shale at 70°C are equally striking. Adamantanes, like methane in thermal stability and reactivity, become highly reactive on carbon surfaces [30]. Solid hydrocarbons in source rocks are not ordinary hydrocarbons. They possess receptors, perhaps catalytic, that react with light hydrocarbons at low temperatures generating solid hydrocarbons of greater mass. The Miller Heirs field data and the ingestion results reported here make a substantial case for methane and solid hydrocarbons in equilibrium in source rocks. The fact that a general equilibrium also accounts for other properties of light hydrocarbons - natural gas at thermodynamic equilibrium and higher hydrocarbons in constant compositions – makes that case even stronger. The capacity of solid hydrocarbons to ingest gas reported here raises the possibility of substantially more gas in shales than analytically apparent, and far more gas in shale deposits than currently recognized.

Only the question of reversibility and perhaps generation remain. Catalysis would seem a given since methane could not react with solid hydrocarbon at 70°C (Table 1) without catalytic assistance. It is, in my view, extremely unlikely that methane could be found in equilibrium with ethane and propane in reservoir rocks [7] and not have been in equilibrium with solid hydrocarbons in source rocks. The Miller Heirs production curve in Figure 7 suggests that it is and that gas generated from solid hydrocarbons sustains production over time (Figure 8). That possibility becomes near certainty should hydrocarbons produced from source rocks transition from steady state to pre-steady state and those produced from conventional reservoirs do not.

## Competing interests

The author declares that there are no competing interests.

## Acknowledgements

Author thanks Petroleum Habitats and Worldwide Geochemistry for their generous support.
